# Transformational Leadership, Career Adaptability, and Work Behaviors: The Moderating Role of Task Variety

**DOI:** 10.3389/fpsyg.2019.02922

**Published:** 2020-01-10

**Authors:** Yujuan Lan, Zhixia Chen

**Affiliations:** ^1^College of Public Administration, Huazhong University of Science and Technology, Wuhan, China; ^2^Career Development Center, Zhongnan University of Economics and Law, Wuhan, China

**Keywords:** career adaptability, transformational leadership, task variety, task performance, organizational citizenship behavior

## Abstract

Career adaptability is a set of individual resources that benefit one’s sustainable development in his/her lifelong careers, especially in today’s turbulent environment. However, how to foster employees’ career adaptability through managerial strategies and eventually contribute to organizations remains to be studied. Guided by the career construction theory, we posit a moderated mediation model that transformational leadership (TFL) could strengthen employees’ career adaptability and further foster their task performance and organization-directed citizenship behavior (OCBO), with task variety moderating the mediation effect. We conducted a three-wave survey with 558 supervisor-employee dyads to test the overall model. The results validated that career adaptability mediated the links between TFL and task performance as well as OCBO. Furthermore, the mediation effect was stronger for employees who had higher levels of task variety. In short, our study offers the groundwork to understand that employees’ career adaptability can be activated by transformational leaders and is self-regulatory to benefit work behaviors in the task variety context. It enlightens organizations to cultivate employees’ career adaptability in the way of TFL and job design, with the objective of promoting the sustainable development for both the employees and the organizations.

## Introduction

Past decades have witnessed tremendous changes in the nature of careers (Lee et al., 2018). The technology advances diminish employment opportunities and increase career transitions. A growth of organizations entering offshore market requires employees to be more flexible with diverse situations. Additionally, organizations expect employees to take on more work roles, join in different teams, and conduct various new tasks. However, there still exist a great number of enterprises where employees engage in monotonous jobs, adopt simple skills, and have limited career development. Most of these employees are neither prepared for career changes nor creating values for organizations, making the enterprises brittle in response to the turbulent environment. Given these trends, both scholars and practitioners prioritize to develop employees’ career adaptability, which is defined as “a psychosocial construct that denotes an individual’s resources for coping with current and imminent vocational development tasks, occupational transitions and personal traumas in their occupational roles that, to a certain extent, alter their social integration” (Savickas, 2005, 2013). Scholars have established robust links between employees’ career adaptability and favorable outcomes, such as work engagement (Xie et al., 2016), career satisfaction (Karatepe and Olugbade, 2017), and psychological well-being (Zhuang et al., 2018). Therefore, developing employees’ career adaptability is an important investment not only for enterprises to remain competitive and ensure sustainability but also for employees to achieve their career goals.

However, how to foster employees’ career adaptability through managerial strategies and eventually contribute to the organizations remains to be studied. Although there is consensus that both individual characteristics and contextual factors are determinants of career adaptability, empirical studies have ignored the influence of contextual predictors, and even fewer studies have taken leadership into consideration. Given that leadership is an important reference for employees to obtain work information and perceive work characteristics, prior studies largely agree on the virtues of leadership to foster effective functioning in employees. Among all the leadership styles, transformational leadership (TFL) has been proven to effectively influence employees’ psychological resources and eventually boost performances in the changing work environment (Ng, 2017); we propose that TFL could be one of the possible antecedents of employees’ career adaptability and further improve their job performance. Meanwhile, the changing nature of the contemporary business world is well reflected by employees’ task variety in their daily work, which is manifested in the aspects of unpredicted circumstances, diverse tasks, and different clients (Hackman and Oldham, 1976). Empirical evidence has shown that higher task variety could enhance transformational leaders’ motivating efforts and results on employees (e.g., Wu and Wang, 2015). These results pave the way for us to explore the boundary condition for the effects of TFL on employees’ career adaptability and work behaviors.

To address the issue, we draw on career construction theory (CCT). It asserts that contextual factors can shape individuals’ career adaptability, which in turn predicts their vocational outcomes across different contextual transitions (Savickas, 2013). Accordingly, we focus on employees’ career adaptability and examine its antecedent (TFL) and outcomes (employees’ work behaviors), with the boundary condition (employees’ task variety). First, we examine whether TFL reinforces employees’ career adaptability resources. CCT contends that career adaptability resides at the intersection of the person-in-environment and can be activated by factors within the environment (Mcilveen and Midgley, 2015). Furthermore, psychological features provided by the environment are the key factors that trigger the activation of one’s career adaptability (Mischel and Shoda, 1995). This analysis naturally leads us to the TFL literature, which indicates that transformational leaders can both shape tangible environments and enrich employees’ psychological resources (Fernet et al., 2015). Thus, they can stimulate employees’ career adaptability. Second, another point of the CCT is that career adaptability shapes one’s self-regulatory strategies and therefore predicts one’s work behaviors (Johnston, 2016). To ascertain the self-regulation role of career adaptability, we test the mediation effects of employees’ career adaptability on TFL and work behaviors in terms of task performance and organization-directed citizenship behavior (OCBO). Task performance focuses on doing something necessary and useful in the formal system, while OCBO focuses on doing something more to further promote the effective functioning of the organization. Therefore, the two kinds of work behaviors represent the key contributions that the adaptable employees may make to their organizations. Third, extant research has shown that some of the job characteristics may favor the emergence and effectiveness of TFL (Bacha, 2014; Wu and Wang, 2015). High task variety represents a diverse and changeable work characteristic that offers high level of transformational leaders’ managerial discretion and strengthens their connection with their followers (Bakker and Demerouti, 2017). As such, we expect that the supportive leadership might be coupled with task variety to fuel employees’ career adaptability and promote their work behaviors in turn.

**Figure 1:**
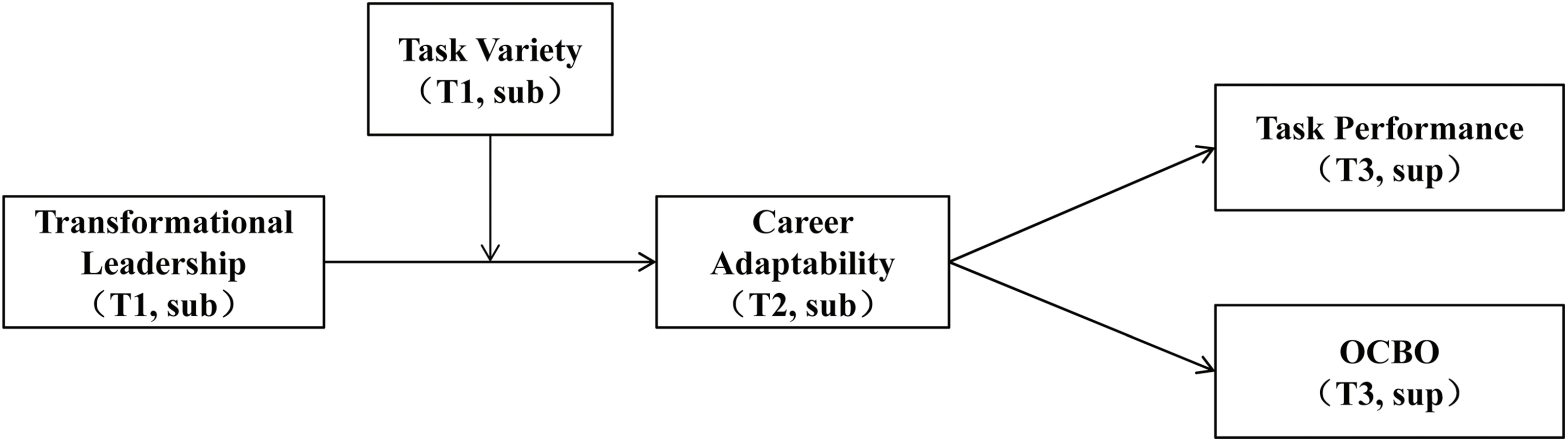
The proposed model. Transformational leadership and task variety, time 1 (T1) rated by subordinates (sub); career adaptability, time 2 (T2) rated by subordinates (sub); task performance and OCBO, time 3 (T3) rated by supervisors (sup).

Overall, the purpose of this study is to provide a comprehensive understanding of the influence of TFL on employees’ career adaptability and work behaviors, with a boundary condition of task variety. We conducted a multisource multiwave survey study to validate our hypotheses. Compared with previous research, this study makes several contributions. First, it expands the CCT by identifying TFL as an antecedent and reveals its mechanism on employees’ career adaptability. Namely, it provides a leadership perspective that few previous literature has adopted. This perspective also complements the practical knowledge of how to cultivate employees’ career adaptability in the organizational context. Second, the current research validates the self-regulation process of career adaptability. By establishing an integrative model, we can explain how to develop career adaptability and utilize it effectively to achieve vocational outcomes, which will extend the application of CCT. Third, testing the moderating role of task variety not only reveals the boundary condition that TFL influences career adaptability and work behaviors but also provides implications that job design will assist in exerting the effectiveness of transformational leaders. Figure 1 depicts the overall theoretical model.

## Theory and Hypotheses

### Career Construction Theory

Previous scholars have recognized two main approaches to conceptualize career adaptability. One is the dispositional approach, which considers career adaptability as a relatively stable and trait-like disposition that differs across individuals (Horst et al., 2017). The other is the situational approach, which suggests that career adaptability can be activated in response to the environment (Karatepe and Olugbade, 2017). Considering our research intention and in response to the call for more contextual predictors of career adaptability (Savickas, 2005, 2013), we adopted the situational approach to outline the causes and consequences of career adaptability. The central construct of CCT is career adaptability, which comprises four subscales: career concern, control, curiosity, and confidence. *Concern* refers to the interest in one’s career aspirations and the acknowledgement of present actions linking to the attainment of future goals. *Control* is characterized by keeping self-disciplined and motivated to achieve one’s career goals. *Curiosity* indicates the openness to new experiences and the exploration or inquiry of career opportunities. *Confidence* reflects one’s belief in one’s ability to solve problems and actualize career goals (Savickas and Porfeli, 2012).

CCT highlights two crucial features of career adaptability. First, unlike stable dispositional traits, career adaptability resources vary in different situations and can be developed in the workplace (Zacher, 2016). Second, career adaptability resources are the self-regulation strengths and capacities for a person to cope with vocational tasks and fit into the surroundings (Savickas and Porfeli, 2012). Based on these features, CCT has proven to be a useful framework to understand the role of TFL intervention in employees’ career adaptability and work behaviors (Guan et al., 2016; Pan et al., 2018).

### Transformational Leadership and Career Adaptability

TFL entails behaviors that alter employees’ standards and values and mobilizes them to achieve organizational goals that exceed their individual interests (Burns, 1978; Bass, 1985). This leadership style generally consists of four dimensions: idealized influence, inspirational motivation, individualized consideration, and intellectual stimulation. *Idealized influence* involves behaving in charismatic ways that inspire followers to identify with them. *Inspirational motivation* refers to articulating visions that are appealing to followers. *Individualized consideration* entails attending to followers’ needs, acting as mentors or coaches, and listening to followers’ concerns. *Intellectual stimulation* indicates that leaders challenge assumptions, take risks, and solicit followers’ ideas (Podsakoff et al., 1990). Li et al. (2015) tested the TFL model in the Chinese context and identified four dimensions of Chinese TFL: *charisma*, *vision articulation*, *individualized consideration*, and *moral modeling*. The first three dimensions have similar definitions with their counterparts in the classic TFL model, while the fourth one (*moral modeling*) is defined as setting examples for the followers who identify with the values the leaders espouse. They found that Chinese transformational leaders are characterized more by *moral modeling* than by *intellectual stimulation*. We propose that transformational leaders positively affect employees’ career adaptability on the basis of the following concerns.

First, from a career construction perspective, career adaptability can be shaped by vocational developmental tasks, and it can also be fostered by communications that explain these tasks (Savickas, 2005). Transformational leaders proactively seek and identify changes that are needed in the organization (Bass, 1985), and they function to shape the work environment with developmental tasks (Bass and Avolio, 1990). They also reframe organizational environment to allow employees to be fully engaged with different aspects of their jobs (Ng, 2017). As such, employees have more chances to connect themselves to new situations in the workplace, which can trigger employees’ adaptability resources (Wang et al., 2017). In addition, transformational leaders are crucial references for employees to interpret their work experiences. They assist the employees’ adaptation process by using different communication skills (O’Connell et al., 2008). They demonstrate the meanings and values of vocational tasks and changes and also discuss difficulties and strategies with the followers to achieve organizational goals. In this case, employees have less inclination to resist change (Oreg and Berson, 2011) and more commitment to change (Herold et al., 2008). Employees can get tangible resources from the workplace created by transformational leaders.

Moreover, CCT also states that adaptability resources are close to the view of psychological capital, which is defined as an individual’s positive psychological state of development (Luthans et al., 2007). TFL inspires employees and strengthens their psychological resources in response to work demands (Gooty et al., 2009). Specifically, transformational leaders have a sense of the future, based on which they articulate visions and provide organizational missions to convince employees of the necessity of embracing the future and make preparations in the present (career concern) (Fernet et al., 2015). By providing practical guidance and feedback, they improve followers’ knowledge, skills, and abilities to solve problems and make decisions on their own (career control) (Bacha, 2014). Moreover, transformational leaders are good at inspiring followers to innovate, challenge traditional assumptions, and be curious about their surroundings (career curiosity) (Khalili, 2016). In addition, by identifying and developing employees’ strengths, transformational leaders can stimulate employees’ self-efficacy and self-awareness of their full potential (career confidence) (Aggarwal and Krishnan, 2013). Moreover, several studies revealed that TFL is an effective predictor of the employees’ adaptability (Nemanich and Keller, 2007; Wang et al., 2017). Griffin et al. (2010) also found that leaders’ visions predicted an increase in adaptability for individuals.

Taken together, transformational leaders can both shape the tangible work environment and enrich employees’ psychological resources, so they can directly enhance employees’ career adaptability. As a result, we proposed the following:

*Hypothesis 1: TFL is positively related to employees’ career adaptability*.

### Career Adaptability, Task Performance, and Organization-Directed Citizenship Behavior

Task performance is recognized by the formal reward system and directly related to the organization’s requirements and tasks (Williams and Anderson, 1991; Harrison et al., 2006). In contrast, organizational citizenship behavior (OCB) is an individual’s discretionary behavior that helps to enhance organizational effectiveness but is not a formal requirement (Organ, 2015). There are two categories of OCB: OCBI (Individual-directed OCB) and OCBO. OCBI are behaviors that benefit specific individuals at work (e.g., helps others who are absent); OCBO are behaviors that directly benefit the organization as a whole (e.g., volunteers for special tasks). Prior studies suggest that the distinction between OCBI and OCBO is important because they have different antecedents and should be treated as separate categories (Williams and Anderson, 1991; Lin et al., 2017). Since we focus on whether the adaptable employees’ work behaviors can make contributions to their organizations, we consider OCBO as a desirable reaction. Task performance and OCBO are fundamental to an organization’s ability to compete in the turbulent environment and to meet the changing needs of the market (Zhou and Hoever, 2014).

On the one hand, we hypothesize that career adaptability has positive relations with employees’ task performance. Guo et al. (2014) found that career concern and curiosity are functional in setting goals, while career control and confidence are functional in achieving goals. Career concern and curiosity help one to explore new experiences, make sense of present work, and prepare for tasks and challenges that foster task performance (Rudolph et al., 2017). In addition, career confidence and control serve to regulate employees’ work behaviors, shape strategic responses, and believe in employees’ ability to improve their task performance to turn organizational goals into reality (Zacher, 2016).

On the other hand, adaptable employees do not limit their efforts to meet the requirements of the assigned tasks and official targets. One of the motivations for adaptable employees to conduct OCBO is to better fit into the organization (Xu and Yu, 2019). OCBOs, such as volunteering for unforeseen tasks, are usually salient behaviors that can be well observed and are desirable in the eyes of supervisors (Wang et al., 2019). Career adaptability could help employees to synthesize their self-concepts into the collective organization, so they are motivated to take on off-duty activities on behalf of the organization. Another motivation to conduct OCBO is that adaptable employees have surplus capabilities and underused self-management resources to broaden task boundaries and engage in extra activities for their organization than those who have restricted career adaptability resources (Neureiter and Traut-Mattausch, 2016). Wang et al. (2017) found that adaptable employees may expand task boundary and relational surroundings that facilitate the organization to better deal with change. On the basis of the above analyses, we assumed the following:

*Hypothesis 2: Career adaptability is positively related to task performance (H2a) and OCBO (H2b)*.

### The Mediating Role of Career Adaptability

We further propose that employees’ career adaptability bridges the link between TFL and two positive work behaviors. Although transformational leaders could activate employees’ career adaptability resources, they cannot directly control their followers’ adaptive behaviors. Namely, the followers have to be self-managed to reach their desired goals (Dust et al., 2014; Steinmann et al., 2018). In CCT, career adaptability denotes an individual’s self-regulation resources which can be activated by environmental factors and direct the individual to be self-regulatory in performing adaptive behaviors (Savickas and Porfeli, 2012). As a supportive workplace reference, TFL will fill in employees’ career adaptability resources. In turn, career adaptability enables employees to take personal responsibility to address work tasks and achieve better performance. In other words, career adaptability helps employees translate transformational leaders’ management into meaningful reflections. Wang et al. (2017) showed that employees’ adaptability mediated the link between TFL and job crafting. Karatepe and Olugbade (2017) found that supervisor support influenced career satisfaction and turnover intentions through career adaptability. Guan et al. (2016) also reported that individuals with organizational support utilize career adaptability resources to achieve career success. Additionally, Zacher (2016) found that daily career adaptability fluctuates in line with job demands and predicts daily task performance. Moreover, the positive benefits of the supportive leadership enjoyed by employees obligate them to reciprocate with prosocial behaviors that contribute to the organization (i.e., OCBO) (Lin et al., 2017). These findings provide evidence indicating that career adaptability could be the underlying mechanism linking TFL to employees’ positive work behaviors. Accordingly, we proposed the following:

*Hypothesis 3: Career adaptability mediates the relationship between TFL and employees’ task performance (H3a) and OCBO (H3b)*.

### The Moderating Role of Task Variety

Task variety is a job characteristic that involves performing diverse tasks by using different skills and talents and frequently encountering exceptional circumstances that require flexibility (Dean and Snell, 1991). Previous studies showed that specific context could strengthen or weaken the influence of TFL on their followers (Bass and Avolio, 1990; Khalili, 2016). For example, Wu and Wang (2015) found that the effect of TFL on team proactivity is stronger when the team task variety is higher. Besides, TFL is more likely to emerge and take effect when the environment is unstable, uncertain, or turbulent (Leuteritz et al., 2017). Accordingly, we expect that if the job design offers some degree of variety, TFL might be more supportive to promote employees’ career adaptability.

To address the interplay of leadership and task variety on employees’ career adaptability and work behaviors, we adopted motivational mechanism of TFL. It asserts that transformational leaders motivate employees to “be willing” to dedicate effort to and “feel capable” about doing well in novel or unexpected tasks, both of which contribute to better performance (Ng, 2017). Among the four dimensions of career adaptability, career concern and curiosity refer to the awareness and willingness to explore career opportunities, while career confidence and control refer to the motivation and capability to achieve career goals (Savickas and Porfeli, 2012). In other words, employees’ career adaptability resources are the manifestation of their willingness and capability to deal with changing tasks and aim at achieving adaptation goals. Consistent with Ng (2017), we propose that TFL strengthens employees’ career adaptability when facing task variety (Griffin et al., 2010).

When task variety is high, employees are exposed to novel situations which make them uncertain about the meaning of job demands and unable to solve new problems (Bakker and Demerouti, 2017). In this case, TFL has stronger impact on employees’ career adaptability. On the one hand, transformational leaders construct task variety as necessary adaptation and clarify the meaning of employees’ experiences in diverse tasks (Gang et al., 2011). They encourage employees to see task variety as opportunities for learning, development, and growth (Sosik et al., 2004). Reciprocally, employees are more likely to make sense of task variety and more willing to take initiative at work (Dust et al., 2014). On the other hand, task variety offers opportunity for transformational leaders to cultivate employees’ work skills and increase their confidence in their capabilities (Bacha, 2014). Transformational leaders provide role models in facing task variety so that employees can learn vicariously from their leaders (Khalili, 2016). They enable new ways of thinking, encourage innovative problem solving, and provide coaching for subordinates. Thus, they promote employees’ self-control over task variety (Li et al., 2015). In total, TFL is desirable to motivate employees’ career adaptability in response to high task variety (Aggarwal and Krishnan, 2013).

When task variety is low, employees are normative and driven by the fixed working rules, procedure, skills, or clients (Wu and Wang, 2015). They are able to work more independently and less motivated to respond to the interventions by their leaders. In other words, the impact of TFL on employees’ career adaptability is weakened (Breevaart et al., 2016). For one thing, jobs with low variety have well-defined tasks and set standards to guarantee consistency and effectiveness (Narayanan et al., 2009). Both leaders and followers are expected to follow specific guidelines, which are explicitly illustrated policies, procedures, and responsibilities (Guan et al., 2016). It may limit transformational leaders’ efforts to make sense of employees’ work and to incent employees to change (Piccolo and Colquitt, 2006). For another, low task variety provides fewer opportunities for employees to adopt different skills and develop their capabilities (Pan et al., 2018). Employees follow routine methods to work and are less dependent on transformational leaders’ effort at motivating their self-efficacy and capabilities (Leuteritz et al., 2017). Thus, the functioning of TFL on employees’ career adaptability is restrained when task variety is low (Aggarwal and Krishnan, 2013). Taken together, we propose the following hypothesis:

*Hypothesis 4: Task variety moderates the relationship between TFL and employees’ career adaptability, such that this relationship is stronger when task variety is high rather than low*.

As transformational leaders instill career adaptability resources through motivating employees’ willingness and capabilities in high task variety, employees have stronger internal motives to proactively deliver better task performance and be good organizational citizens (Manaf and Latif, 2014). As a result, higher task variety provides a platform that enhances transformational leaders’ efforts to develop employees’ career adaptability and positive work behaviors. On the contrary, the effectiveness of TFL in enhancing employees’ career adaptability is attenuated when task variety is low. In turn, employees may be less activated to deliver better task performance and to voluntarily engage in OCBO (Steinmann et al., 2018). Hence, we propose the following:

*Hypothesis 5: Task variety moderates the mediation effect of career adaptability between TFL and task performance (H5a) as well as OCBO (H5b). Specifically, the mediation effect is stronger at high levels rather than at low levels of task variety*.

## Methods

### Sample and Procedure

The survey was carried out off-line in nine organizations in the cities of Beijing, Guangzhou, Chengdu, Shanghai, and Wuhan, which are located respectively in the north, south, west, east, and middle of China. In addition, these organizations have comprehensive departments and positions which can increase the representativeness of professions. In total, 1,420 employees voluntarily participated in the survey and were assured of anonymity. They are from different departments and positions and have different organizational tenures. To avoid common method bias (Podsakoff et al., 2003), we coded supervisor-employee dyads with the assistants of the human resources (HR) departments and adopted a three-wave data collection with a 1-month interval between each investigation.

At time 1, employees reported their demographic information and completed questionnaires on their leaders’ TFL and their own task variety. We distributed 1,420 questionnaires and retrieved 1,060 valid questionnaires from employees, with a response rate of 74.6%. At time 2, the targeted 1,060 employees were asked to rate their career adaptability, and 755 of them provided valid replies, yielding a response rate of 71.2%. At time 3, the corresponding supervisors were invited to evaluate the targeted 755 employees’ task performance and OCBO, resulting in 558 valid answers and a response rate of 73.9%. The questionnaires were filled out by all participants separately without discussion. Among the employees, 52.7% were female, and 77.8% held a bachelor’s degree or above. On average, their age was 32.7, and their organization tenure was 5.6 years. Among the supervisors, 44.3% were female, and 79.4% held a bachelor’s degree or above. On average, their age was 36.2, and their position tenure was 7.6 years.

### Measures

The scales of the English version were subjected to Brislin’s (1970) back-translation procedure for the assurance of linguistic equivalence. Before the formal survey, we conducted a pilot study to ensure all the scales are valid measures. Results show that all the scales have adequate to excellent reliabilities and validities.

#### Transformational Leadership (Time 1)

We used the Chinese edition of the TFL questionnaire (TLQ) (Li and Shi, 2005), which was revised from the international edition for Chinese culture (Bass and Avolio, 1990; Podsakoff et al., 1990). The 26-item scale contains four dimensions: inspirational motivation, individualized consideration, idealized influence, and moral modeling. A sample item was “My supervisor facilitates the acceptance of group goals.” Employees rated their leaders on a 5-point Likert scale ranging from “strongly disagree” (1) to “strongly agree” (5). The Cronbach’s alpha for this scale was 0.85.

#### Task Variety (Time 1)

We adopted a seven-item scale from Dean and Snell (1991). Employees self-reported the extent to which a job involved different tasks, clients, methods, and procedures in a 5-point Likert scale. Sample items include “How much opportunity do members have in this unit to do a number of different things?” (1 = very little, 3 = a moderate amount, 5 = a great deal). The Cronbach’s alpha was 0.87.

#### Career Adaptability (Time 2)

We used the Career Adapt-Ability Scale (CAAS), China form (Hou et al., 2012), which was translated from the international version (Savickas and Porfeli, 2012). The 24-item scale is divided equally into four subscales that measure the adaptability resources of concern, control, curiosity, and confidence, each with six items. Sample items included “Looking for opportunities to grow as a person” and “Thinking about what my future will be like.” Employees rated their responses from “not strong” (1) to “strongest” (5). The Cronbach’s alpha was 0.91.

#### Task Performance (Time 3)

Supervisors were asked to evaluate their employees’ task performance using the seven-item scale developed by Williams and Anderson (1991). Sample items included “This employee adequately completes the assigned duties” and “performs tasks that are expected of him/her.” Supervisors rated on a 5-point Likert scale ranging from “strongly disagree” (1) to “strongly agree” (5). The reliability coefficient for this scale was 0.88.

#### Organization-Directed Citizenship Behavior (Time 3)

Supervisors also completed the eight-item measure of employees’ OCBO published by Lee and Allen (2002). Sample items included “This employee shows pride when representing the organization in public” and “expresses loyalty toward the organization.” Supervisors rated on a 5-point Likert scale ranging from “strongly disagree” (1) to “strongly agree” (5). The reliability coefficient for this scale was 0.89.

#### Control Variables

To exclude the potential influence of demographic factors and test the unique contribution of TFL as a predictor of career adaptability and job performance, employees’ demographic variables were controlled, including their gender (0 = male, 1 = female), age (years), organization tenure (years), and education level (1 = below bachelor’s degree, 2 = bachelor, 3 = master, 4 = above master), because of their potential effects on career adaptability and job performance (Horst et al., 2017).

### Analytical Approach

Before hypothesis testing, we constructed five competing models and conducted confirmatory factor analysis (CFA) with AMOS 20.0. As TFL and career adaptability each have over 20 observed indicators, item parceling is a recommended procedure, as it has several advantages over item-level CFA estimation (Little et al., 2002; Piccolo and Colquitt, 2006). Hence, four item parcels were created for the overall-level career adaptability and TFL, in accordance with each one’s four dimensions.

To test the hypotheses, we adopted Hayes’s PROCESS Macro for SPSS to measure the indirect effects for mediation and moderated mediation (Edwards and Lambert, 2007; Hayes, 2013). PROCESS analyses were based on 5,000 bootstrap samples, and the coefficients were significant when the construction of 95% bias-corrected confidence intervals (CIs) did not include zero.

## Results

### Preliminary Analyses

The CFA results in Table 1 showed that the five-factor model fitted the data better than did the alternative models [*χ*^2^(395) = 624.65, *p* < 0.05, comparative fit index (CFI) = 0.97, root mean square error of approximation (RMSEA) = 0.03]. Table 2 demonstrates the convergent validity of the measured constructs. The standardized factor loadings of each scale were above the acceptable threshold of 0.60 (*p* < 0.05). The composite reliabilities (CRs) of the five measured constructs were all above the minimum acceptable value of 0.70 (Hair et al., 2009). The average variance extracted (AVE) values of the five constructs were all above 0.50 (Fornell and Larcker, 1981). Furthermore, the square roots of the AVE values of any two constructs were greater than their correlation estimates, further supporting the adequate discriminant validity of all measured constructs (Hair et al., 2009) (see Table 3). Given the results, we concluded that the scales were measuring distinctive constructs and continued to test the proposed hypotheses.

**Table 1:** Summary of model fit indexes.

Factor structure	*χ*^2^	*df*	*χ*^2^*/df*	CFI	TLI	RMSEA	AIC	BIC	SRMR	PNFI	PCFI
Five-factor model	624.65	395	1.58	0.97	0.97	0.03	764.65	1067.35	0.03	0.84	0.88
Four-factor model	1729.37	399	4.65	0.81	0.80	0.08	1988.72	2274.13	0.09	0.71	0.75
Three-factor model	2761.15	402	6.87	0.70	0.68	0.10	2887.15	3159.58	0.13	0.62	0.65
Two-factor model	4343.82	404	10.75	0.50	0.46	0.13	4465.82	4729.61	0.19	0.44	0.46
One-factor model	4925.44	405	12.16	0.42	0.38	0.14	5045.44	5304.90	0.15	0.38	0.39

*TFL, transformational leadership; TV, task variety; CA, career adaptability; TP, task performance; OCBO, organization-directed citizenship behavior. Five-factor model: TFL, TV, CA, OCBO, and TP; four-factor model: TFL, TV, CA, and (OCBO + TP); three-factor model: (TFL + TV), CA, and (OCBO + TP); two-factor model: (TFL + TV + CA) and (OCBO + TP); one-factor model: (TFL + TV + CA + OCBO + TP). CFI, comparative fit index; TLI, Tucker-Lewis index; RMSEA, root mean square error of approximation; AIC, Akaike information criterion; BIC, Bayesian information criterion; SRMR, standardized root mean square residual; PNFI, parsimony-adjusted normed fit index; PCFI, parsimony-adjusted comparative fit index*.

**Table 2:** Reliabilities and AVE values.

Constructs	Factor loadings	Composite reliability	AVE
1. TFL	0.71–0.80	0.83	0.56
2. Task variety	0.68–0.73	0.87	0.50
3. Career adaptability	0.83–0.84	0.90	0.70
4. OCBO	0.68–0.78	0.89	0.50
5. Task performance	0.65–0.75	0.88	0.51

*All the factor loadings are significant. AVE, average variance extracted; TFL, transformational leadership; OCBO, organization-directed citizenship behavior*.

**Table 3:** Descriptive statistics and correlations.

Constructs	Mean	SD	Correlations								
			1	2	3	4	5	6	7	8	9
1. Age	32.73	7.24	NA								
2. Organization tenure	5.56	3.62	0.62^**^	NA							
3. Gender	0.54	0.50	−0.05	0.01	NA						
4. Education	2.14	0.81	−0.08	−0.15^**^	0.04	NA					
5. TFL	3.54	0.48	0.09^*^	0.06	−0.01	0.09^*^	**0.75**				
6. Task variety	2.97	0.76	0.02	0.04	0.70	0.08	−0.04	**0.71**			
7. Career adaptability	3.87	0.49	0.07	0.08	0.08	0.01	0.35^**^	−0.10^*^	**0.84**		
8. OCBO	3.52	0.65	0.11^**^	0.13^**^	0.09^*^	0.01	0.21^**^	0.36^**^	0.39^**^	**0.71**	
9. Task performance	3.82	0.64	0.12^**^	0.12^**^	0.08	−0.01	0.36^**^	0.07	0.42^**^	0.36^**^	**0.72**

*TFL, transformational leadership; OCBO, organization-directed citizenship behavior; NA, not applicable*.

*The diagonal row of intercorrelations contains the square roots of the constructs’ average variance extracted (AVE) values*.

* *p < 0.05*;

** *p < 0.01*.

### Descriptive Statistics

Table 3 shows the means, standard deviations, and correlations. TFL was positively related to career adaptability (*r* = 0.35, *p* < 0.01), OCBO (*r* = 0.21, *p* < 0.01), and task performance (*r* = 0.36, *p* < 0.01). In addition, career adaptability was significantly related to task variety (*r* = −0.10, *p* < 0.05), OCBO (*r* = 0.39, *p* < 0.01), and task performance (*r* = 0.42, *p* < 0.01).

### Hypotheses Testing

Hypotheses 1 and 2 predicted that TFL was positively related with career adaptability and that career adaptability was positively related with task performance and OCBO. As shown in Table 4, after the demographic variables were controlled for, TFL is positively related to career adaptability [*β* = 0.39, *p* < 0.001, 95% CI (0.32, 0.47)], and career adaptability is positively related to task performance [*β* = 0.43, *p* < 0.001, 95% CI (0.33, 0.53)] and OCBO [*β* = 0.49, *p* < 0.001, 95% CI (0.38, 0.59)]. Therefore, Hypotheses 1, 2a, and 2b were supported.

**Table 4:** Examining the moderated mediation model.

Variables	Career adaptability				Task performance				OCBO			
	Coeff	SE	95% LL	95% UL	Coeff	SE	95% LL	95% UL	Coeff	SE	95% LL	95% UL
Constant	3.70^***^	0.10	3.49	3.90	1.98^***^	0.24	1.50	2.46	1.38^***^	0.25	0.88	1.88
Age	0.01	0.01	−0.01	0.01	0.01	0.00	−0.01	0.01	0.01	0.01	−0.01	0.01
Tenure	0.01	0.01	−0.01	0.02	0.01	0.01	−0.01	0.03	0.01^*^	0.01	0.01	0.03
Gender	0.08^*^	0.04	0.01	0.15	0.08	0.05	−0.02	0.17	0.09	0.05	−0.01	0.19
Education	0.01	0.02	−0.03	0.06	−0.02	0.03	−0.08	0.04	0.01	0.03	−0.05	0.08
TFL	0.39^***^	0.04	0.32	0.47	0.32^***^	0.05	0.22	0.43	0.10	0.06	−0.01	0.21
Career adaptability					0.43^***^	0.05	0.33	0.53	0.49^***^	0.05	0.38	0.59
Task variety	−0.09^***^	0.02	−0.13	−0.04								
TFL ^*^ task variety	0.43^***^	0.04	0.34	0.51								
*R*^2^	0.25				0.24				0.18			
*F*	26.58^***^				29.00^***^				20.57^***^			

*N = 558. Bootstrap sample size = 5,000*.

*We try to control the effects of employees’ industry and position in the hypothesis testing, yet they do not impact our results and conclusions. So they were not included in the final model for the sake of parsimony. OCBO, organization-directed citizenship behavior; UL, upper limit; LL, lower limit; TFL, transformational leadership*.

* *p < 0.05*;

*** *p < 0.001*.

Hypothesis 3 proposed that employees’ career adaptability mediated the relationship between TFL and work behaviors. The results showed that the indirect effects of TFL through career adaptability on task performance [estimate = 0.15, *p* < 0.001, 95% CI (0.08, 0.23)] and on OCBO [estimate = 0.17, *p* < 0.001, 95% CI (0.10, 0.25)] were both significant. Thus, Hypotheses 3a and 3b were supported. In addition to the indirect effects, the direct effect of TFL on task performance was still significant [estimate = 0.32, *p* < 0.001, 95% CI (0.22, 0.43)], but the direct effect of TFL on OCBO was not significant [estimate = 0.10, *p* > 0.05, 95% CI (-0.01, 0.21)] (see Table 4). Consequently, the results indicated that career adaptability fully mediated the effect of TFL on OCBO but partially mediated the effect of TFL on task performance.

**Figure 2:**
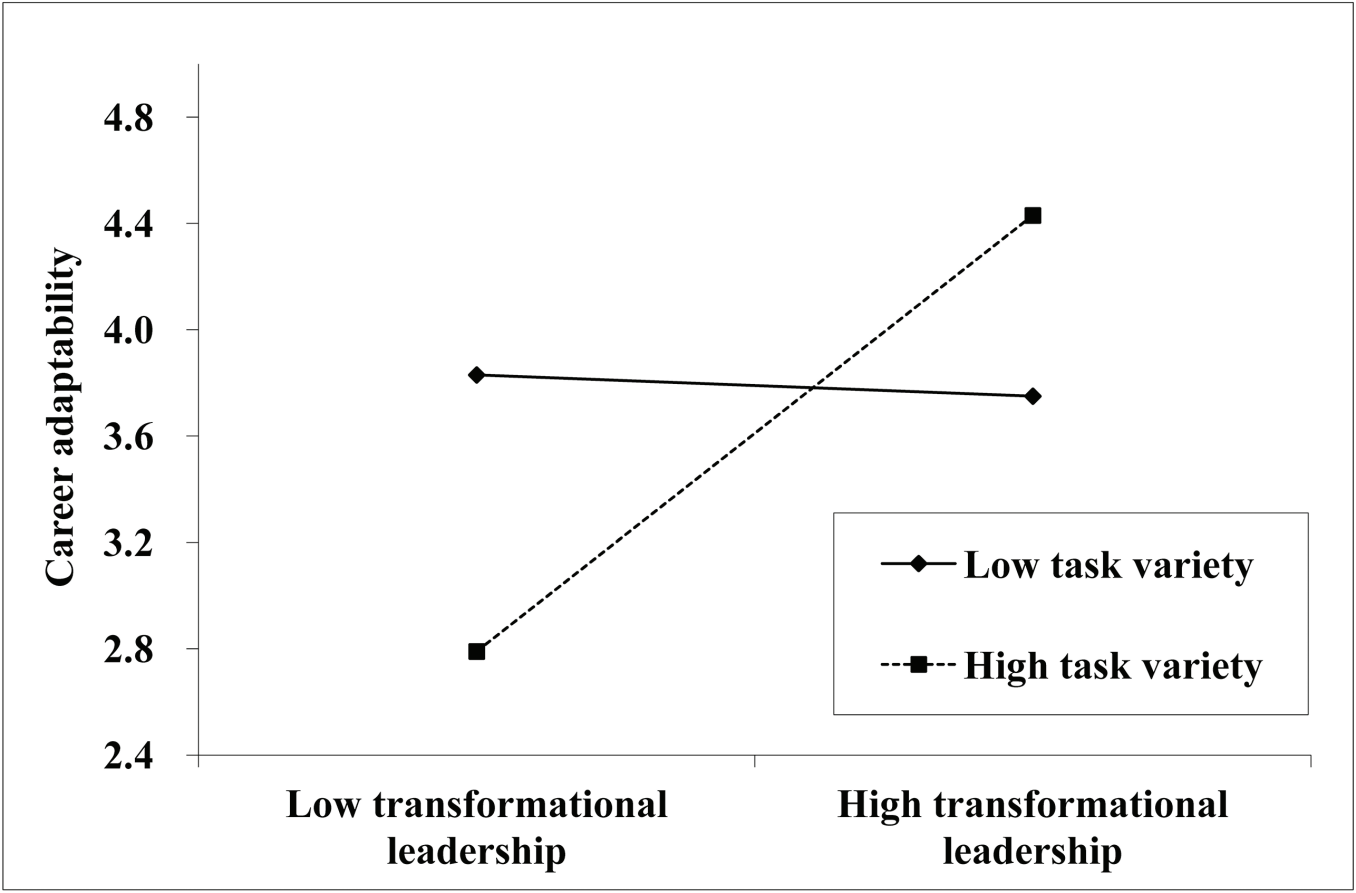
Effect of interaction of transformational leadership and task variety on career adaptability.

Hypothesis 4 postulated the moderation effect of task variety on the link between TFL and employees’ career adaptability. Before analysis, the interaction terms of TFL and task variety were mean-centered (Aiken and West, 1991). The results showed that the interaction between TFL and task variety had a significantly positive effect on career adaptability [*β* = 0.43, *p* < 0.001, 95% CI (0.34, 0.51)]. We further plotted the interactive effect at one standard deviation above and below the mean of task variety, which could represent the high versus low levels of the moderator (Aiken and West, 1991). As shown in Figure 2, the slope was steeper when task variety was high than when it was low. Simple slope tests revealed that when task variety was high, the effect of TFL on career adaptability was significantly positive (*β* = 0.82, *p* < 0.001). However, when task variety was low, such effect was not significant (*β* = −0.04, *p* > 0.05). Accordingly, Hypothesis 4 was supported.

Hypothesis 5 depicted the moderated mediation effect. The indirect effect of TFL on task performance and OCBO through career adaptability was compared between one standard deviation (SD) above and below the mean of task variety. As shown in Table 5, when task variety was low, the indirect effects of TFL on task performance [estimate = 0.03, *p* > 0.05, 95% CI (−0.03, 0.09)] and on OCBO [estimate = 0.03, *p* > 0.05, 95% CI (−0.03, 0.10)] were not significant, as both of the 95% CIs included zero. When task variety was high, the indirect effects of TFL on task performance [estimate = 0.31, *p* < 0.01, 95% CI (0.19, 0.43)] and on OCBO [estimate = 0.35, *p* < 0.01, 95% CI (0.23, 0.47)] were higher and significant. Overall, the conditional indirect effect is 0.18 on task performance [*p* < 0.01, 95% CI (0.10, 0.27)] and 0.21 on OCBO [*p* < 0.01, 95% CI (0.12, 0.30)]. Hence, Hypotheses 5a and 5b were supported.

**Table 5:** Conditional indirect effect as a function of task variety.

Moderator = task variety		TFL → career adaptability → task performance				TFL → career adaptability → OCBO			
		Effect	SE	95% LL	95% UL	Effect	SE	95% LL	95% UL
Conditional indirect effect	Low (−1SD)	0.03	0.03	−0.03	0.09	0.03	0.03	−0.03	0.10
	High (+1SD)	0.31^**^	0.06	0.19	0.43	0.35^**^	0.06	0.23	0.47
Moderated mediation effect		0.18^**^	0.04	0.10	0.27	0.21^**^	0.04	0.12	0.30

*N = 558. Bootstrap sample size = 5,000*.

*OCBO, organization-directed citizenship behavior; UL, upper limit; LL, lower limit; TFL, transformational leadership*.

** *p < 0.01*.

## Discussion

This research examined why and when leaders engaging in TFL behaviors affect employees’ career adaptability and positive work behaviors. In particular, a transformational leader will lead a flexible workforce who has the ability to deal with the changing job requirements and exceed the organizational expectations to eventually enhance the organizational competitiveness.

### Theoretical Implications

First, we expand CCT by demonstrating the role of human intervention in facilitating career adaptability, and in particular, we identify TFL as a precursor that supports career adaptability. As past research predominantly focused on the dispositional antecedents of career adaptability, the lack of situational predictors as well as the human intervention of career adaptability has led scholars to doubt whether career adaptability is, as theorized, a dynamic construct that can be developed (Coetzee and Harry, 2014). In accordance with CCT, we view career adaptability as a psychosocial construct, which is a set of accumulated resources gained through social experience and human intervention (Cheung and Jin, 2016). In our study, TFL was found to be one of the effective interventions for employees’ career adaptability. Since transformational leaders both enrich employees’ external resources by shaping the work environment and instill employees’ internal psychological resources, they could facilitate the integration of employees’ work roles and organizational environment. Specifically, transformational leaders create a meaningful work environment for employees to gain insights for their career development. Meanwhile, by inspiring, motivating, and providing individual concern and support, transformational leaders manage to foster employees’ positive psychological resources in relation to career adaptability. Recent studies have tested the effectiveness of some situational factors, including organizational support (Guan et al., 2016) and parental behavior (Guan et al., 2015), in developing ones’ career adaptability. Our study extended these findings from the leadership perspective and further supports the malleability of employees’ career adaptability.

Second, the current study finds that employees’ career adaptability mediates the link between TFL and employees’ task performance and OCBO. By doing so, we validate the self-regulatory nature of career adaptability (Johnston, 2016). Based on CCT, career adaptability serves as self-regulatory capacities, which are motivational and instrumental in taking actions and achieving work goals (Savickas, 2013). It not only enables employees to take responsibility for their work behaviors by showing self-discipline but also helps them translate organizational management into meaningful reflections. To be specific, we find that career adaptability fully mediates the TFL and OCBO link. This filtering mechanism corroborates the previous research by Purvanova et al. (2006). They posited that TFL altered followers’ perceptions on their work duties and roles, thus indirectly influencing employees’ tendencies to engage in OCBO. Nasra and Heilbrunn (2015) also found that employees of transformational leaders internalized the goals of the group. Therefore, employees were likely to view leaders’ supportive behaviors as meaningful and consistent with their self-concept, indicating that OCBO could be intrinsically motivated. Unlike the full-mediation effect, career adaptability partly mediates the link between TFL and task performance. Similarly, Manaf and Latif (2014) found that the adaptability cultural trait partly mediated TFL and technical staffs’ job performances. Bass (1985) explained that transformational leaders inspired followers to perform beyond expectations so that the followers required both managerial resources and individual resources to accomplish tasks. Our work also enriches the extant empirical research that examines the underlying mechanisms of TFL’s influence on employees’ performance, showing that employees’ career adaptability could be one of the valid pathways. Employees get actual benefits from TFL in terms of career adaptability resources, which help them become self-navigated to boost positive work behaviors in order to achieve organizational goals.

Third, we provide evidence that task variety moderates the impacts of TFL on employees’ career adaptability and positive work behaviors, which contributes to the TFL literature. As stated above, those who are both willing and able to address changing conditions are expected to demonstrate more positive work behaviors (Savickas, 2005). On the basis of the motivational mechanism of TFL, transformational leaders are crucial to increasing employees’ career adaptability resources, because they motivate employees to be willing and able to deal with diverse new tasks and capitalize on the changing opportunities, both of which contribute to favorable performance and OCBO in turn (Cullen et al., 2014). Consequently, the positive effects of TFL on employees’ career adaptability and work behaviors are strengthened by task variety. In general, high task variety, as opposed to low task variety, offers a higher level of transformational leaders’ managerial discretion in motivating the followers, provides more opportunities for employees’ career exploration, and therefore strengthens the connections between transformational leaders and their followers. Our findings not only reveal the boundary condition of transformational leaders’ impact on employees’ career adaptability but also validate previous findings that the emergence and effectiveness of TFL depend on organizational context (Leuteritz et al., 2017). Additionally, research in career adaptability has seldom considered or examined the interactive effects of leader’s intervention and job characteristic, which are two kinds of typical organizational factors in the development of employees’ career adaptability.

### Practical Implications

Our research findings provide several managerial implications. First, we show that employees’ career adaptability is developable in the workplace and that TFL could be one of the valid interventions. Therefore, we recommend that leaders adopt TFL behaviors to foster the employees’ career adaptability, which could boost their employability and eventually benefit the organization. Furthermore, in line with Savickas’ (2005) assertion, transformational leaders could attempt to promote employees’ career concern by orientation exercises, control by decisional training, confidence by self-efficacy building, and curiosity by information-seeking tasks. Those proactive career management strategies will enable employees to boost overall career adaptability.

Second, we validate the self-regulatory function of career adaptability in the process of organizational management and work-related behaviors. In the turbulent business world, both scholars and practitioners emphasize individual responsibility for career development (Rudolph et al., 2017). Our research reassures organizational concern that individual career adaptability not only contributes to the effectiveness of the organizational mandate but also navigates self-development to gain favorable outcomes in the workplace. Namely, to improve task performance and OCBO, it is important for leaders to increase employees’ self-management resources and is also essential for employees to utilize these resources in response to different situations (Johnston, 2016).

Third, we suggest organizations redesign job characteristics by embracing task variety. Under this circumstance, transformational leaders may exert effectiveness on improving employees’ career adaptability by making sense of their work, as well as motivating employees to cope with work variations. Hence, we recommend that organizations take a more active approach in establishing and maintaining environments with variety, changes, transformations, and innovations to some extent, as well as redesigning job characteristics to enhance the development of employees’ career adaptability, task performance, and OCBO, which ultimately contribute to the organization.

### Limitations and Future Research

The limitations of our study should be acknowledged. First, this study was conducted in five typical cities in China, which would limit the generalization of our findings to other cultures. Considering this, the nine organizations that we chose have cross-culture business, and employees have more or less intercultural experiences in the workplace. Nonetheless, we suggest testing our model in different cultures and regions to validate the findings.

Second, we adopted self-report and supervisor-report approaches to measure all the variables. Accordingly, it is hard to rule out subjective factors, such as the supervisor-subordinate relationship and social desirability, which may bias the measurement. To improve the measurement, further research could take observable behaviors as alternatives (e.g., longitude design with daily work record) to reflect the employees’ actual reactions.

Third, for theoretical reasons, we only examined situational factors including TFL and task variety as the antecedent and moderator of career adaptability. However, it is likely that both individual and contextual variables can shape career adaptability and further predict vocational outcomes. Future studies can expand the present work by testing the interactive effects of more organizational factors with individual characteristics on employee’s career adaptability and to make a comprehensive understanding of the career construction mechanism.

## Conclusions

In summary, our study provides empirical evidence of a connection between TFL and job performance *via* the indirect effect of employees’ career adaptability, with the conditional factor of task variety. These findings supplement research on the CCT and the TFL theory, as well as organizational management practices. For employees, developing career adaptability not only improves self-development but also benefits their work outcomes. For leaders, the effectiveness of TFL can be manifested by improving employees’ career adaptability. For organizations, job redesign with variety could strengthen the link between leaders’ management and employees’ adaptability and further benefit overall performances.

## Data Availability Statement

The datasets generated for this study are available on request to the corresponding author.

## Ethics Statement

The studies involving human participants were reviewed and approved by the ethics committee of Huazhong University of Science and Technology. The patients/participants provided their written informed consent to participate in this study.

## Author Contributions

YL, the first author, presented the idea and wrote the main part of the manuscript. ZC, the corresponding author, built the structure of the article and is in charge of the whole project.

### Conflict of Interest

The authors declare that the research was conducted in the absence of any commercial or financial relationships that could be construed as a potential conflict of interest.
